# Effect of fed-batch and chemostat cultivation processes of *C. glutamicum* CP for L-leucine production

**DOI:** 10.1080/21655979.2021.1874693

**Published:** 2021-01-20

**Authors:** Yufu Zhang, Haibo Xiong, Zhichao Chen, Yunpeng Fu, Qingyang Xu, Ning Chen

**Affiliations:** College of Biotechnology, Tianjin University of Science & Technology, Tianjin, P. R. China; Key Laboratory of Industrial Fermentation Microbiology, Tianjin University of Science & Technology, Ministry of Education, Tianjin, P. R. China; Tianjin Key Laboratory of Industrial Microbiology, Tianjin University of Science & Technology, Tianjin, P. R. China

**Keywords:** L-leucine, L-alanine, *Corynebacterium glutamicum*, chemostat, fed-batch

## Abstract

Most of the current industrial processes for L-leucine production are based on fermentation, usually in fed-batch operation mode. Although the culture technology has advanced in recent decades, the process still has significant drawbacks. To solve these problems, we investigated the effects of chemostat culture conditions on the production of L-leucine by *Corynebacterium glutamicum* CP. The dilution rate, the nitrogen source, and the carbon–nitrogen ratio of the medium were optimized. With the addition of ammonium acetate to the chemostat medium, the initial C/N ratio was adjusted to 57.6, and the L-leucine titer reached the highest level at the optimal dilution rate of 0.04 h^−1^. Compared with fed-batch culture, the L-leucine titer was reduced (from 53.0 to 24.8 g L^−1^), but the yield from glucose was increased by 10.0% (from 0.30 to 0.33 mol mol^−1^) and productivity was increased by 58.3% (from 1.2 to 1.9 g L^−1^ h^−1^). Moreover, the titer of the by-product L-alanine was significantly reduced (from 8.9 to 0.8 g L^−1^). In addition, gene expression levels and activity of key enzymes in the synthesis of L-leucine and L-alanine were analyzed to explain the difference of production performance between chemostat culture and fed-batch culture. The results indicate that chemostat culture has great potential to increase the industrial production of L-leucine compared to current fed-batch approaches.

## Graphical abstract

**Figure:**
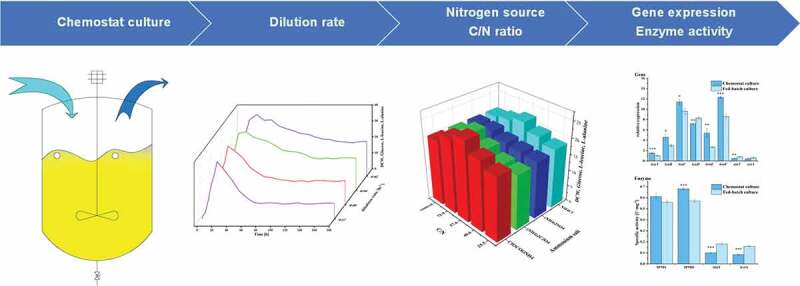

## Introduction

As one of the branched-chain amino acids (BCAAs), L-leucine is an essential amino acid that cannot be synthesized by mammals [1]. Its applications range from animal feed additives to ingredients in cosmetics, and specialty nutrients in pharmaceutical or medical preparations [2,3]. L-leucine is also used as an infusion additive to improve the nutritional status of patients with liver disease [4]. It was reported that L-leucine plays an important role in stimulating muscle protein synthesis and maintaining glucose homeostasis [4,5]. The production methods of L-leucine include extraction from protein hydrolyzates, chemical synthesis, and microbial fermentation [6].

Due to the mild conditions, high yield, as well as economic and environmental advantages, microbial fermentation has become one of the most attractive processes for the commercial production of L-leucine [6,7]. Nowadays, the fed-batch culture mode is usually adopted in the production of L-leucine by microbial fermentation. Although the fermentation process is advanced and widely used, it still has some drawbacks [8–11], such as requiring highly trained experienced operators due to the uncertain timing of feed addition during the fermentation. Currently, the control points for the production are only empirically determined or based on static parameters of several tests. It is difficult to simultaneously meet the needs of microbial growth and product synthesis, resulting in shortening of the high-productivity period of amino acid synthesis and reducing the total output.

In chemostat culture, the nutrient solution is fed continuously at a constant rate, and a mixed solution containing microorganisms, nutrients, and metabolites is withdrawn at the same rate. The relatively stable environment provided by the chemostat can effectively extend the stationary phase of the microorganisms in batch culture or fed-batch culture, keep the quality of the target metabolites relatively stable, and also improve the productivity of metabolites and equipment utilization. Besides, various parameters in the continuous cultivation of microorganisms tend to have fixed values, which is convenient for automatic management and control. Compared with the fed-batch culture method in which the environment is constantly changing, the chemostat culture method has unique advantages.

In the laboratory, chemostat culture can not only be used to analyze the growth, reproduction, and metabolic pattern of a certain microbial strain, but it can also be employed for the selection of species that have excellent genotype and phenotype [12–15]. Compared with the batch culture, chemostat culture has more advantages in the adaptive laboratory evolution [16]. Previous studies have provided insight into the potential use of chemostat culture to improve the production performance of bacteria. The volumetric and specific productivity of recombinant lipase B by *Pichia pastoris* under continuous mode were 1.5 and 3 times greater than the fed-batch mode, respectively [17]. Chemostat culture is also widely used in industrial processes such as the production of yeast cells or single-cell protein, as well as the fermentation of chemical products such as ethanol, lactic acid, and acetone. The chemostat culture mode has drawn the attention of many researchers and has become one of the most active research topics in bioprocess engineering [18]. Moreover, the chemostat culture has the characteristics of strong operability and steady measurable parameters, and mathematical models for predicting experimental results are easier to build [19,20].

There were still some defects in the production of L-leucine by *C. glutamicum* CP under the fed-batch culture. Firstly, when the L-leucine titer surpassed 30 g L^−1^, precipitated crystals formed a large amount of foam. Secondly, the production of L-leucine was performed in a growth-coupled process, so that the productivity of L-leucine decreased significantly in the stationary phase. Finally, in the late stage of fermentation production, more byproduct L-alanine was accumulated. The main objective of this work was to solve these problems. Therefore, we adopt the chemostat culture and optimized the culture conditions to improve L-leucine production performance and decrease the byproduct yield. Furthermore, we analyzed gene expression level and enzyme activity of *C. glutamicum* CP under chemostat culture and fed-batch culture. This study demonstrates that chemostat culture has great potential for improving the industrial production of L-leucine.

## Materials and methods

### Strain and culture media

*Corynebacterium glutamicum* CP, a leucine-producing strain, was obtained through multiple rounds of random mutation and screening and was deposited with the China General Microbiological Culture Collection Center under the accession number CGMCC 11425. *C. glutamicum* CP is resistant to α-thiazole alanine, α-aminobutyric acid, β-hydroxyleucine, sulfaguanidine, and rifampicin and auxotrophic for methionine and isoleucine. The entire genome of *C. glutamicum* CP was sequenced and assembled to a 3,342,897 bp chromosome (Genbank Accession No. CP012194.1) and a 4,447 bp endogenous unnamed plasmid (Genbank Accession No. CP012412.2) [21].

The medium used for seed culture contained (per liter): 40 g glucose, 40 g corn steep liquor (CSL), 2 g KH_2_PO_4_ · 12H_2_O, 2 g MgSO_4_ · 7H_2_O, 5 mg FeSO_4_ · 7H_2_O, 0.2 g L-methionine, 0.3 g L-isoleucine, 0.5 mg biotin, and 0.2 mg thiamine.

The fermentation medium for fed-batch or chemostat culture consisted of (per liter): 100 g glucose, 10 g CSL, 5 mL soybean meal hydrolyzate[22], 2 g KH_2_PO_4_ · 12H_2_O, 3 g MgSO_4_ · 7H_2_O, 30 mg MnSO_4_·H_2_O, 30 mg FeSO_4_ · 7H_2_O, 0.2 g L-methionine, 0.3 g L-isoleucine, 2 g L-glutamic acid, 0.3 mg biotin, and 0.3 mg thiamine. All media were adjusted to pH 7.0–7.2 using NaOH (80 g L^−1^). Vitamins, such as biotin, thiamine, were sterilized by filters (0.2 μm in diameter), before which was added the complete medium. The reagents were purchased from SINOPHARM (China).

Ammonium acetate (CH_3_COONH_4_), ammonium oxalate ((NH_4_)_2_C_2_O_4_), ammonium sulfate ((NH_4_)_2_SO_4_), and ammonium chloride (NH_4_Cl) were individually added to the chemostat culture feed medium, and their concentration was adjusted to change the initial C/N ratio of the medium, as shown in Table 1. The rest of the ingredients and culture conditions remained unchanged.

**Table 1.** Composition of chemostat culture feed media with different C/N ratios

C/N ratio (mol/mol)	(NH_4_)_2_SO_4_ (g L^−1^)	(NH_4_)_2_C_2_O_4_ (g L^−1^)	CH_3_COONH_4_ (g L^−1^)	NH_4_Cl (g L^−1^)
99.2	0	0	0	0
72.9	0.5	0.5	0.6	0.4
57.6	1.0	1.0	1.2	0.8
40.6	2.0	1.9	2.5	1.6
25.5	4.0	3.9	5.1	3.2

### Fermentation protocols

#### Fed-batch cultivation

Fed-batch cultivations were performed in a 30-L fermenter (Baoxing Bio-Engineering, Shanghai, China) with an initial working volume of 14 L. The seed culture was transferred to the bioreactors at the 15% (v/v) inoculum size. The culture was aerated at an initial rate of 0.6 vvm (volume per volume and minute) with filtered air and kept at 32°C. The stirrer speed was set to 300 rpm and was increased up to 800 rpm to keep the dissolved oxygen saturation (DO) at 20–30% of the initial value. The pH was kept constant at 7.0–7.2 by the automatic addition of ammonium hydroxide (25%, v/v). Antifoam was added to the bioreactor to prevent foam formation when necessary. When the concentration of glucose in the medium was reduced to 10 g L^−1^, feed solution (80% glucose, w/v) was added to maintain the concentration at approximately 5 g L^−1^.

### Chemostat culture

The chemostat was composed of three main components [23]: fermenter, liquid supply system, and overflow device. The inoculation, cultivation temperature, DO, and pH were consistent with the fed-batch cultivation. The stationary phase of the fed-batch culture was indicated by no further increase of biomass. The chemostat culture was started with the addition of chemostat culture feed medium at a constant rate, and the culture broth was removed at the same rate to keep the working volume constant. Cultures were grown at 32°C with the air flowrate of 1 vvm. By changing the dilution rate, nitrogen source, and initial C/N ratio of the chemostat medium, the chemostat culture process was optimized. The four nominal dilution rates selected were based on previous literature [17,24]. The C/N ratio referred to molar ratio of carbon and total Kjeldhal nitrogen. The steady state was achieved after continuous operation for at least three residence time. The early stage of chemostat culture was consistent with that of fed-batch culture, so we compared gene expression level and key enzyme activity in the stationary phases during the fed-batch process and the steady state of the chemostat process. L-leucine and L-alanine titer were compared at the end of two fermentation processes. The production performances, such as productivity and yield, were compared throughout the fermentation processes.

D =$\frac{F}{V}$, (D, dilution rate, F: feeding rate L h^−1^, V: bioreactors working volume, L)

### Analytical methods

Fermentation broth samples comprising 5 mL were taken every 4 h for analysis. Cell growth was determined by detecting changes in dry cell weight (DCW) as described previously [25].

The detection and quantification of glucose in the culture supernatants were performed using an SBA-40E immobilized enzyme biosensor (Biology Institute of Shandong Academy of Sciences, Jinan, China). Amino acid concentrations were measured by high-performance liquid chromatography (HPLC) using a LC20AT system (Shimadzu, Kyoto, Japan) equipped with an Agilent ZORBAX Eclipse AA column (4.6 mm × 150 mm, 5 µm; Agilent Technologies, Palo Alto, CA, USA) with UV detection (360 nm). Acetate buffered acetonitrile was used as the mobile phase at a flow rate of 1 mL min^-1^ [26]. All data were measured in triplicate.

### Quantitative real-time PCR for mRNA quantification

Total RNA from the collected bacteria was extracted using the Eastep Super Total RNA Extraction Kit (Promega, Shanghai, China), according to the manufacturer’s instructions. Then, the total RNA was reverse-transcribed into cDNA using PrimeScript^TM^ RT Master Mix (TaKaRa, Dalian, China). RT-PCR was conducted on a StepOne Real-Time PCR System (Thermo-Fisher Scientific, USA) using TB Green® Premix Ex Taq™ II (Tli RNase H Plus; TaKaRa, Dalian, China) and the primers listed in Table 2, as described before [26]. The transcriptional level of 16S ribosomal RNA was used as an internal reference.

**Table 2.** Primers used for quantitative real-time PCR

Primer No.	Target gene		Sequence (5ʹ-3ʹ)	Amplicon size (bp)
1	*leuA*	Forward	AGGTTGAGTTCTCCACCGTTGTC	124
2		Reverse	ATCTGCTCAACTGGTGCGGT	
3	*leuB*	Forward	TTGCTGTTATTGGTGGAGATGGTAT	147
4		Reverse	AAGGTCATAATCGGTGGTCTCG	
5	*leuC*	Forward	ATGACCAGCCCCGTGGAGAAC	105
6		Reverse	GTCGGGCTCGCCGTTTTCT	
7	*leuD*	Forward	ATCCCAGCCGTCTACCTCAAG	141
8		Reverse	CGAATCGTGAGGAGAAGACAGC	
9	*brnE*	Forward	TTGCGATTACAGTGGTGGCG	119
10		Reverse	CCTGCTGGCATCCACATCG	
11	*brnF*	Forward	CGGCGTGGCGACTTATCTCA	159
12		Reverse	GGCAGGAATCCAAAGTCAGCGT	
13	*alaT*	Forward	GTCTATCCAAGGCATACCGCG	134
14		Reverse	TGGGACATTTGGGCAGAGTC	
15	*avtA*	Forward	CAGCCCAGGAAACCCAACG	136
16		Reverse	TGCCAGCGGACGACCAAAGC	
17	16S rRNA	Forward	CGATACGGGCATAACTTGA	181
18		Reverse	GTTTACGGCATGGACTACC	

### Preparation of crude enzyme solution and determination of enzyme activity

The crude enzyme solution was prepared to determine the enzyme activities of isopropylmalate synthase (IPMS), 3-isopropylmalate dehydratase (IPMD), alanine aminotransferase AlaT, and alanine aminotransferase AvtA. The cells were collected by centrifugation (8000 g, 10 min, 4°C), and washed with 50 mM Tris-HCl. Then, the resuspended cells were sonicated and centrifuged (Beckman Coulter Avanti J-26S XP, 40000 g, 30 min, 4°C) to remove cell debris. The supernatant constituted the crude enzyme solution. The protein concentration was determined using the BCA Protein Assay Kit (Solarbio, Beijing, China). The protein concentration and enzyme activity were measured in triplicate.

The IPMS enzyme activity was determined by detecting coenzyme A formation using Ellmann’s reagent [27]. One unit of enzyme activity was defined as the amount of enzyme which converts 1 µmol of α-isopropylmalate per min under the optimal reaction conditions of the assay.

IPMD enzyme activity was determined by detecting the production of the reaction intermediate α-isopropylmaleate [27]. One unit of enzyme activity was defined as the amount of enzyme which converts 1 µmol α-isopropyl maleate per min under the optimal reaction conditions of the assay.

The AlaT/AvtA enzyme activity was determined by detecting the amount of L-alanine produced with L-glutamic acid or L-valine as the amino donor [28]. One unit of enzyme activity was defined as the amount of enzyme which converts 1 µmol of L-alanine per min under the optimal reaction conditions of the assay.

### Statistical analysis

The data obtained were subjected to statistical analysis using SPSS version 18.0 (SPSS Inc., Chicago, USA). All the data were expressed as mean ± standard deviation of three replicates. Student’s t-test was used to compare fed-batch and chemostat cultivation, and differences with p values≤0.05 were considered statistically significant.

## Results and discussion

Due to fast-growing market demand of L-leucine, modified production methods are required. Furthermore, there are still some challenges in L-leucine production using fed-batch culture. The chemostat culture pattern was adopted to improve the L-leucine production performance. Some parameters, such as dilution rate, nitrogen source, and C/N ratio, were optimized. Then, we analyzed gene expression levels and activity of key enzymes in the stationary phases during the fed-batch process and the steady state of the chemostat process. L-leucine fermentative processes by different culture methods were compared in the last section.

### Fed-batch cultivation of C. glutamicum CP

**Figure 1.**
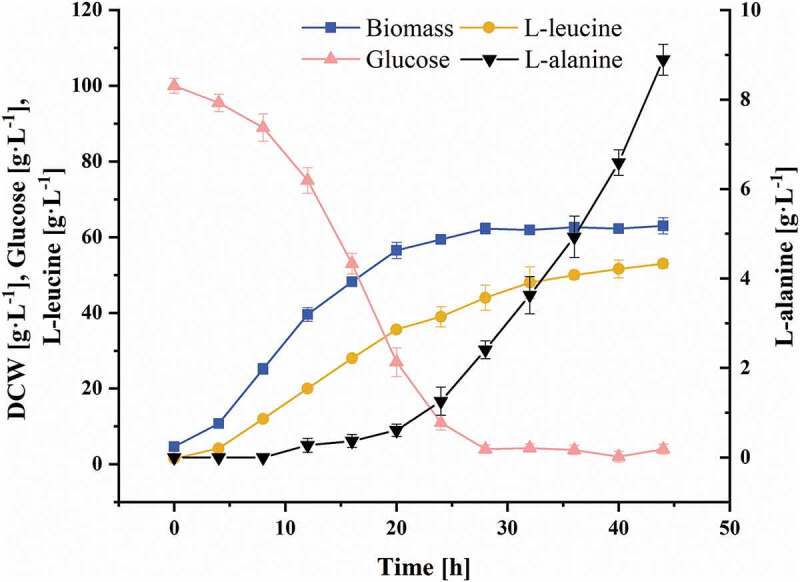
The growth, product/byproduct generation, and glucose consumption profiles of *C. glutamicum* CP in fed-batch culture

The strain *C. glutamicum* CP was characterized in the fed-batch cultivations under controlled conditions, and the fermentation period was extended to 44 h. The fermentation parameters are shown in Figure 1. The production of L-leucine was performed in a growth-coupled process. When *C. glutamicum* CP entered the exponential phase, L-leucine also entered the rapid accumulation phase. After 24 hours of fermentation, *C. glutamicum* CP entered the stationary phase, and the DCW was maintained at about 60 g L^−1^. According to the glucose consumption rate of *C. glutamicum* CP, the glucose addition rate was adjusted to maintain the residual glucose concentration in the fermentation broth at about 5 g L^−1^. When the fermentation broth contains excess glucose, it leads to the Crabtree effect [29], which inhibits the growth of bacteria, increases the generation of byproducts, and decreases the product yield [30]. At the end of the fermentation, the L-leucine titer reached 53.0 ± 1.2 g L^−1^. Due to the strong hydrophobicity of L-leucine, its solubility is only 24.3 g L^−1^ [27], and the fermentation broth became supersaturated after 20 h, leading to the formation of a large amount of precipitated L-leucine crystals. When measured after heating and dilution, the L-leucine titer reached 35.6 ± 0.6 g L^−1^. A large amount of foam formed under the influence of stirring, and it overflowed out from the exhaust port in spite of the antifoam. This condition not only led to the loss of a certain amount of cells and product, but also increased the risk of contamination. The fed-batch process required continuous supplementation of glucose solution, which increased the bioreactor working volume and made the fermentation operation more difficult to control. Since the consumption of nutrients in the culture medium, the accumulation of harmful metabolites, and the decline of the transfer rate of nutrients and dissolved oxygen in the medium due to severe foaming, the productivity of L-leucine decreased significantly. The average productivity of L-leucine was 1.6 g L^−1^ h^−1^ during 0–24 h of the fed-batch process, and the by-product L-alanine titer was 1.3 ± 0.3 g L^−1^. However, during 24–44 h, the average productivity of L-leucine was only 0.7 g L^−1^ h^−1^, while the L-alanine titer was as high as 8.9 ± 0.3 g L^−1^ at later time points of the fed-batch process. To solve these problems, after 20 hours of the fed-batch process, chemostat culture was started at an appropriate dilution rate.

### Effect of dilution rate on chemostat culture of C. glutamicum CP

**Figure 2.**
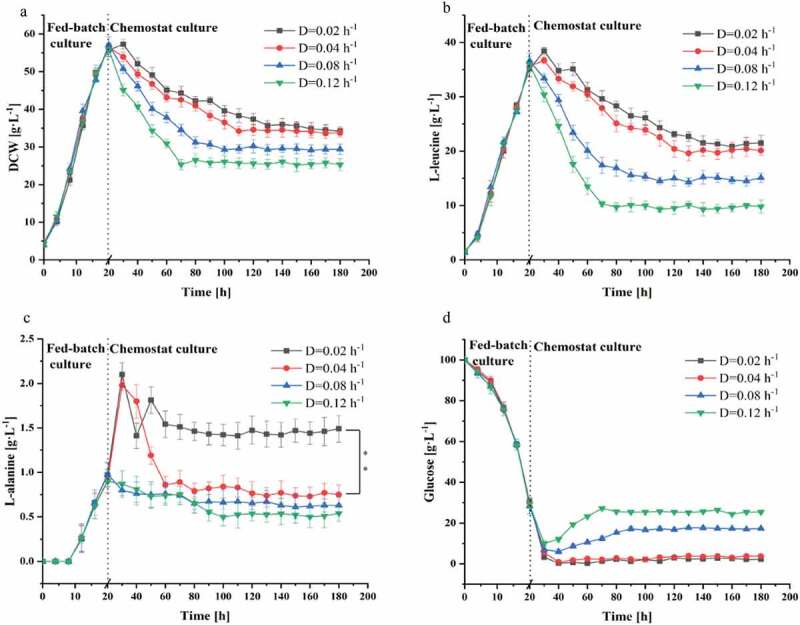
Effect of dilution rate on the chemostat culture of *C. glutamicum* CP. (a) Biomass, (b) L-leucine, (c) L-alanine, (d) Glucose. Data are presented as means ± standard deviations from three independent experiments. *p ≤ 0.05, **p ≤ 0.01, ***p ≤ 0.001

A chemostat culture for the continuous production of L-leucine was started when L-alanine titer was lower than 1 g L^−1^ at 20 hours of the fed-batch culture. Since the culture is at the steady state, the growth rate of the organisms equals the dilution rate [20]. The influence of the dilution rate on L-leucine production by *C. glutamicum* CP was studied. As shown in Figure 2, four dilution rates, including 0.02, 0.04, 0.08, and 0.12 h^−1^, were investigated in the chemostat culture process. As the dilution rate increased, the biomass concentration of *C. glutamicum* CP decreased remarkably, but there was no significant difference in the DCW at the dilution rate of 0.04 and 0.02 h^−1^. The biomass concentration of the steady state remained virtually constant at three different dilution rates which were lower than the maximum specific growth rate [17]. The L-leucine titer at the dilution rate of 0.04 and 0.02 h^−1^ was similar in the steady state, but the L-alanine titer was higher at a lower dilution rate (0.02 h^−1^) than at other dilution rates (Figure 2(c)). The glucose in the outlet flow increased slightly with increasing dilution rate (Figure 2(d)), because the nutrients were washed out of the bioreactor before utilized. The effect of the dilution rate on L-leucine production was examined, and the optimal dilution rate for chemostat culture was determined to be 0.04 h^−1^. Compared to fed-batch operation, chemostat cultivation of microorganisms offers the advantages of constant environmental conditions for biological systems, which can be highly sensitive to process variations [31]. The dilution rate is a critical process parameter of continuous production processes. Kang et al. optimized the growth and lipid production by *Nannochloropsis salina* transformants in continuous culture with variable dilution rates and feed nitrogen concentration [32]. Moreover, Gerritzen et al. improved the productivity of outer membrane vesicles by optimizing the dilution rate in a certain range [33].

### Effects of different ammonium salts and C/N ratios on chemostat culture of C. glutamicum CP

**Figure 3.**
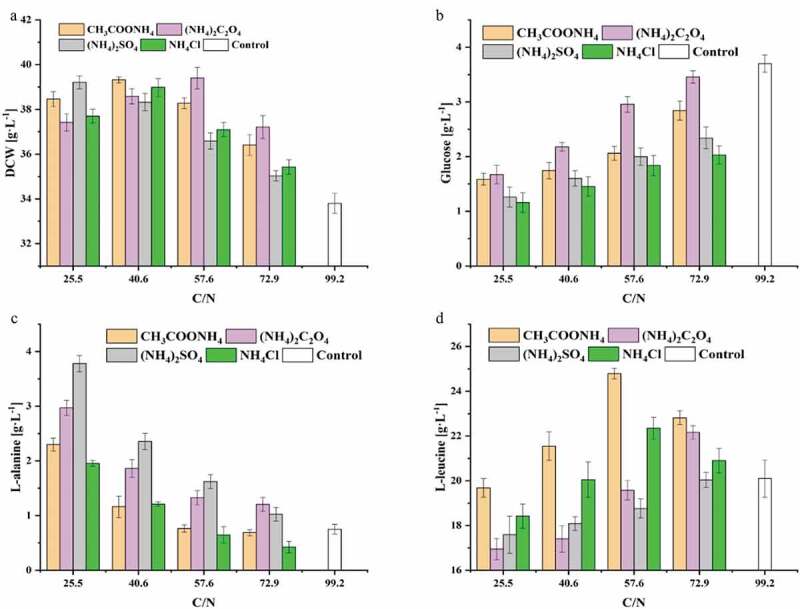
The effect of nitrogen source and C/N ratio on the chemostat culture of *C. glutamicum* CP in the steady state. (a) Biomass, (b) L-leucine, (c) L-alanine, (d) Glucose

Many studies investigated the influence of the type and concentration of carbon and nitrogen sources, as well as the C/N ratio on the fermentation process, since the carbon and nitrogen sources play an important role in the growth of microorganisms. A too high or too low C/N ratio has negative effects on the growth of microorganisms or the synthesis of products. By changing the feeding rate of glucose and ammonium hydroxide to change the C/N ratio in the medium, 102 g L^−1^ L-threonine was obtained in a 5-L fermenter, which was then successfully scaled up in a 500-L fermenter (89 g L^−1^) [34]. A C/N ratio of 5.39 encouraged the proliferation of *Acetobacter xylinum* NUST4.2, while at a C/N ratio of 6.31, metabolic flux analysis showed that 20.96% of glucose was converted into the byproduct bacterial cellulose [35]. Moreover, Ryu et al. optimized the medium for the continuous culture of *Paenibacillus kribbensis* CU01, and the concentration of fusaricidin was increased 28 times compared with the previous reports [36]. In order to investigate the effects of ammonium salt types and the C/N ratio on the production of L-leucine in chemostat cultivation at a dilution rate of 0.04 h^−1^, CH_3_COONH_4_, (NH_4_)_2_C_2_O_4_, (NH_4_)_2_SO_4_, and NH_4_Cl, were added individually to the chemostat medium, and each ammonium salt was set to four concentrations corresponding to C/N ratios of 25.5, 40.6, 57.6, and 72.9. When the C/N ratio was 99.2, no ammonium salt was added. The results are shown in Figure 3.

The biomass of *C. glutamicum* CP increased significantly when ammonium salts were added to the chemostat medium. With (NH_4_)_2_C_2_O_4_ was added at a C/N ratio of 57.6, the DCW of *C. glutamicum* CP in the steady state reached its maximum of 39.4 ± 0.5 g L^−1^, representing a 16.6% increase compared to no ammonium salt addition (Figure 3(a)). However, as the C/N ratio increased, the biomass in the chemostat culture gradually decreased with the addition of (NH_4_)_2_SO_4_. An increased C/N ratio means a decrease in nitrogen content in the medium, leading to slower proliferation of *C glutamicum* CP. In addition, the unbalance nutrient status of the higher C/N ratio increases the osmotic pressure of the culture media, which is detrimental to the growth of *C glutamicum* CP. The maximum value of the biomass in the stable phase was reached with an intermediate C/N ratio with ammonium salts other than sulfate. Due to the addition of ammonium salts, *C. glutamicum* CP grew vigorously, and glucose consumption also increased. The feed medium was supplemented with (NH_4_)_2_SO_4_, and the residual glucose concentration was significantly lower than that of the medium with added (NH_4_)_2_C_2_O_4_ or CH_3_COONH_4_(Figure 3(b)). The reason may be that part of the oxalate and acetate ions were used by *C. glutamicum* CP, reducing the consumption of the substrate. The concentration of L-leucine during the steady state was lower when the medium was supplemented with (NH_4_)_2_SO_4_ or (NH_4_)_2_C_2_O_4_, and higher with CH_3_COONH_4_ or NH_4_Cl. Furthermore, the byproduct titer of L-alanine indicated the opposite trend (Figure 3(c)). Pyruvate is the end product of glycolysis, and there is only one step in the conversion of pyruvate to L-alanine. We thought the excessive ammonium ions affected the carbon metabolic flow, leading to more L-alanine production. When the C/N ratio was 57.6, the concentration of L-leucine reached its maximum, and the concentration of L-leucine in the culture media with added CH_3_COONH_4_ and NH_4_Cl respectively reached 24.8 ± 0.2 and 22.4 ± 0.5 g L^−1^. Compared with no added ammonium salt, the L-leucine titer respectively increased by 22.3% (p < 0.01) and 11.4% (p < 0.05) (Figure 3(d)). However, when (NH_4_)_2_SO_4_ or (NH_4_)_2_C_2_O_4_ was added to the medium, leucine titer in the steady-state was lower than that of the control. With few addition of (NH_4_)_2_C_2_O_4_ (C/N ratio of 72.9), the L-leucine titer respectively increased by 10.2% (p < 0.05) compared with the control. The possible reason was that with the addition of these two ammonium salts, the concentration of ammonium ions in the medium increases, resulting in the decrease of L-leucine production. In summary, when the industrial strain *C. glutamicum* CP was grown in chemostat culture at a dilution rate of 0.04 h^−1^ with the addition of 1.2 g L^−1^ of CH_3_COONH_4_ to the medium (C/N ratio of 57.6), the production of L-leucine reached the highest value in this study. In addition to CH_3_COONH_4_ as a nitrogen source, we speculated that acetic acid ions in the medium may be absorbed and utilized by *C. glutamicum* CP to produce acetyl-CoA which was one of the substrates of the IPMS and thus promote the synthesis of L-leucine. In *C. glutamicum*, the availability of acetic acid was mainly through a two-step enzyme reaction of acetate kinase (AK) and phosphotransacetylase (PTA) [37]. Furthermore, CH_3_COONH_4_ was found to play a dual function in acetone-butanol-ethanol (ABE) fermentation by *Clostridium acetobutylicum*, as it effectively improves ABE fermentation when it is used as the sole nitrogen source but significantly decreases the fermentation performance in the presence of soybean meal [38].

### Expression of genes and key enzyme activities during chemostat culture of C. glutamicum CP

**Figure 4.**
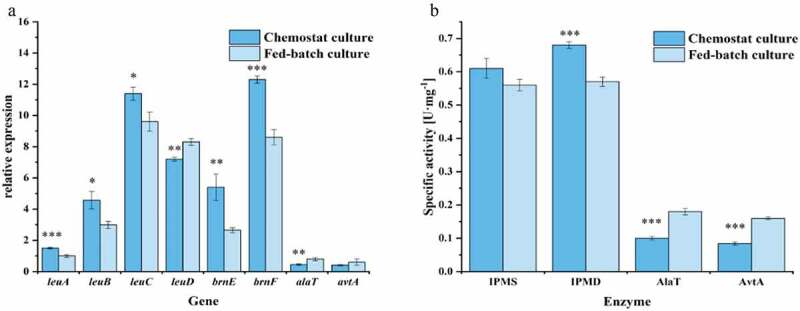
Comparison of gene expression levels and activities of enzymes related to the synthesis of L-leucine and L-alanine. The expression levels of the genes were measured using fluorescence quantitative RT-PCR from RNA extracted in the stationary phases during the fed-batch process and the steady state of the chemostat process. Specific activity of IPMS, IPMD, AlaT, and AvtA in crude extracts of *C. glutamicum* CP were determined, respectively. Data are presented as means ± standard deviations from three independent experiments. *p ≤ 0.05, **p ≤ 0.01, ***p ≤ 0.001

The *leuABCD* genes are specific for the synthesis of L-leucine, compared with other BCAAs. The *leuA* gene encodes IPMS, which is the rate-limiting enzyme and is subject to feedback inhibition by L-leucine. The *leuCD* genes encode IPMD, and *leuB* encodes 3-isopropylmalate dehydrogenase (IPMDH) [39]. The synthesis of the byproduct L-alanine is related to two genes/enzymes, whereby AlaT converts pyruvate to L-alanine in a glutamate-dependent reaction, and AvtA is able to convert pyruvate to L-alanine at the expense of L-valine [28]. In addition, the accumulation of intracellular L-leucine can weaken the transcription of related genes or induce feedback inhibition of key enzymes, which affects the subsequent synthesis of L-leucine. In *C. glutamicum*, the key L-leucine transporter is BrnEF. It was found that *C. glutamicum* Lrp could bind to the intragenic region between *lrp* and *brnF*, thus activating the expression of the *brnFE* genes when BCAAs and L-methionine accumulate in the cell [40,41]. In the fermentation process, gene transcription and enzyme activity levels directly affect product synthesis. To compare the differences between the two culture methods, bacterial cells from the stationary phase of the fed-batch culture and the steady state of the chemostat culture were collected to analyze the gene expression and enzyme activity levels as shown in Figure 4.

The expression of genes related to L-leucine synthesis under chemostat culture was significantly higher than during the stationary phase of fed-batch culture. Previous studies have shown that *C. glutamicum* AN02 can synthesize L-leucine only when *leuA*^CP^ and *leuBCD* are simultaneously overexpressed [26]. Notably, the expression level of *alaT*, which is related to L-alanine production, was significantly lower than in fed-batch culture, while the expression of *avtA* was not significantly different (Figure 4(a)). The *alaT* gene is a major driver of L-alanine synthesis, and the alanine titer in the exponential growth phase was reduced from 95 to 18 mM by deleting *alaT*, whereas *avtA* deletion decreases the L-alanine concentration only to 76 mM [28]. Under chemostat culture, the expression level of *leuA* was higher, but the IPMS enzyme activity was not significantly different from that of fed-batch culture. Moreover, during fed-batch cultivation, the enzyme activity of IPMD was lower, while the enzyme activities of AlaT and AvtA were higher (Figure 4(b)). By comparing the genomes of *C. glutamicum* ATCC13032 and *C. glutamicum* CP, Ma et al. found that amino acid substitutions significantly increased the activity of the mutant IPMS of *C. glutamicum* CP, which led to an increased supply of the precursor 2-isopropylmalate [26]. This may be the reason why there was no significant difference in the activity of IPMS in the two culture modes. Because chemostat culture can continuously provide fresh culture medium and simultaneously remove harmful metabolites, *C. glutamicum* CP increased the production of L-leucine and reduced the production of L-alanine.

### Differences in the production performance between chemostat culture and fed-batch culture

The *C. glutamicum* CP by fed-batch fermentation produced 53.0 g L^−1^ L-leucine with a yield and productivity of 0.30 mol mol^−1^ glucose and 1.2 g L^−1^ h^−1^, respectively. However, the byproduct titer of L-alanine was 8.9 g L^−1^. In chemostat mode, the L-leucine titer was reduced to 24.8 g L^−1^ in the steady state, but the yield of 0.33 mol mol^−1^ increased by 10.0% in comparison to fed-batch mode. Furthermore, the L-leucine productivity was 1.9 g L^−1^ h^−1^, representing a significant increase of 58.3% over the fed-batch process. Lipovsky et al. stated that the highest solvent yield and productivity were achieved in continuous culture compared to batch and fed-batch systems [42]. Moreover, the byproduct titer of L-alanine was significantly reduced, to only 0.8 g L^−1^. The maximum pathway yield of L-leucine depends on the specific pathway and is calculated from its stoichiometry [43]. L-leucine from glucose via the conventional metabolic pathway can be expressed as:

1.5C_6_H_12_O_6_+ NH_3_ + 2O_2_ = C_6_H_13_NO_2_ + 4H_2_O+3 CO_2_

Thus, the maximum pathway L-leucine yield from glucose is 0.67 mol mol^−1^. There is still great potential for yield improvement. Ulteriorly, the high production performance of L-Leucine requires excellent strains and appropriate fermentation process methods. The traditional L-leucine-producing strains were developed by multiple rounds of traditional random mutation and selection, which had some adverse effects, such as growth retardation and byproduct formation. With in-depth understanding of the synthetic pathways of L-leucine and the feedback regulations involved, genetically defined producers were developed by rational genetic engineering. Highly productive strains can be created employing genetically defined strategies, including strengthening the precursor and coenzyme supply, removing the feedback regulation of key enzymes, improving the transcription of key genes and the activity of key enzymes, blocking or weakening competing pathways, increasing export of production, etc. [3]. Various researches related to the metabolic engineering of microbial cells toward L-leucine have been reported. Recent progress in L-leucine production in both *C. glutamicum* and *E. coli* is summarized in Table 3. This strain *C. glutamicum* MV-LeuF2 produced up to 181 mM L-leucine in the chemically defined medium along with precipitate of L-leucine after 72 h of the fed-batch fermentation [27]. Feng et al. reported that a rational modification of aminotransferase activity improved L-leucine production through optimizing the aminotransferases [44]. L-leucine producing strain *C. glutamicum* WL-14, obtained by rational genetic engineering with mutant bacteria as the starting strain, exhibited the high L-leucine titer of 28.5 g L^-1^ [45]. Currently, L-leucine producing *E. coli* strains were less productive than the *C. glutamicum* strains. Besides, *C. glutamicum* has been recognized as generally regarded as safe (GRAS) strain for the production of L-leucine [45]. Except for *C. glutamicum* MV-LeuF2, most other strains were only tested shake-flasks in the semi-defined culture medium and the production was not scaled up yet. The culture temperature used in this study was 32°C, which was higher than that of other *C. glutamicum*. To our knowledge, this is the first report on the production of L-leucine by *C. glutamicum* in chemostat culture. Although the fermentation performance of *C. glutamicum* CP in chemostat cultivation was better than that of other production strains and production methods, we believed that more work needs to be done to achieve the continuous industrial production of L-leucine. Kopp et al. discussed the current state of *E. coli* fed-batch process technology and its potential for technology transfer within continuous processing [46].

**Table 3.** The representative strains for production of L-leucine

Strain	Relevant characteristics	Media	Culture mode	L-leucine titer g L^−1^		L-alanine titer g L^−1^	yield mol mol^−1^	Productivity g·L^−1^·h^−1^	Reference
*C. glutamicum* CP	Mutation of *ilvBN* and *leuA*	semi-defined culture medium	Fed-batch, glucose 80%, 32 °C, 44 h		53.0 ± 1.2	8.9 ± 0.3	0.30	1.2	This work
*C. glutamicum* CP	Mutation of *ilvBN* and *leuA*	semi-defined culture medium	Chemostat, glucose 10%, 32 °C, 100 h		24.8 ± 0.2	0.8 ± 0.1	0.33	1.9	This work
*C. glutamicum* WL-14	Deletion of *ltbR, ppc, avtA*, insertion of terminator T3 in front of *alaT*, replacement of *ilvBNC* and *leuA* promoter by P*tuf*, pECXK99E-*leuA*	semi-defined culture medium	shake-flasks, glucose 13%, 30 °C, 72 h		28.5 ± 0.4	-	-	-	[45]
*C. glutamicum* XQ-9	L-leucine producing *C. glutamicum* strain created by random mutagenesis	semi-defined culture medium	shake-flasks, glucose 13%, 30 °C, 72 h		23.3 ± 0.2	-	0.26	0.3	[47]
*C. glutamicum* FA-1	pECXK99E-*aspB*, deletion of *ilvE*	semi-defined culture medium	shake-flasks, glucose 13%, 30 °C, 72 h		20.8 ± 0.3	2.38	-	0.289	[44]
*C. glutamicum* MV-LeuF2	Deletion of *avtA*; deletion of *iolR*; deletion of *ltbR*; reduction of *gltA*; introduction of *ilvBN^r^*; integration of *leuA*-B018; replacement of leuA-B018 promoter by P*_tuf_*	chemically defined medium	Fed-batch, glucose 50%, 30 °C, 50 h		24	1.8	0.26	0.4	[27]
*E.coli* K-12 No.103	Resistance of L-leucine	chemically defined medium	shake-flasks, glucose 6%, 32 °C, 48 h		1.7	-	-	0.035	[48]
*E.coli* K-12 No.55	Mutation of *leuA*^r^	chemically defined medium	shake-flasks, glucose 6%, 32 °C, 48 h		5.2	-	-	0.108	[49]
*E.coli* K-12 505	Mutation of *ilvE* (double L-valine and L-isoleucine auxotrophy); overexpression of *tyrB*, pACYC-*tyrB*	semi-defined culture medium	shake-flasks, glucose 6%, 32 °C, 48 h		2.7	-	-	0.038	[50]
*E.coli* 57	Feedback-resistant leuA*; inactivation of *ilvE*; replacement of *sucAB* promoter by P*_tac_*_−21_	defined culture medium	shake-flasks, glucose 6%, 34 °C, 72 h		11.4	-	-	0.158	[51]

## Conclusions

Although the fed-batch process is advanced and widely used, it still has some defects. In this research, the industrial L-leucine production strain *C. glutamicum* CP was used to study the effects of chemostat culture conditions on L-leucine production. Chemostat culture method delivered superior production performance compared to fed-batch culture manner. The results of this study indicate that chemostat culture is a very promising method for the industrial production of L-leucine. However, the byproduct L-alanine still exists in the chemostat culture, so rational genetic engineering modification is further needed to reduce the production of L-alanine.

## Supplementary Material

Supplemental Material

## Highlights

Chemostat culture was firstly used in L-leucine production.

Optimum ammonium salt and C/N ratio were obtained.

Comparison between chemostat and fed-batch culture modes.
