# Supplementary figures and images for: Effect of fed-batch and chemostat cultivation processes of C. glutamicum CP for L-leucine production
Source: Bioengineered. 2021 Jan 20;12(1):426–39. doi: 10.1080/21655979.2021.1874693 (PMC8806242; doi:10.1080/21655979.2021.1874693)

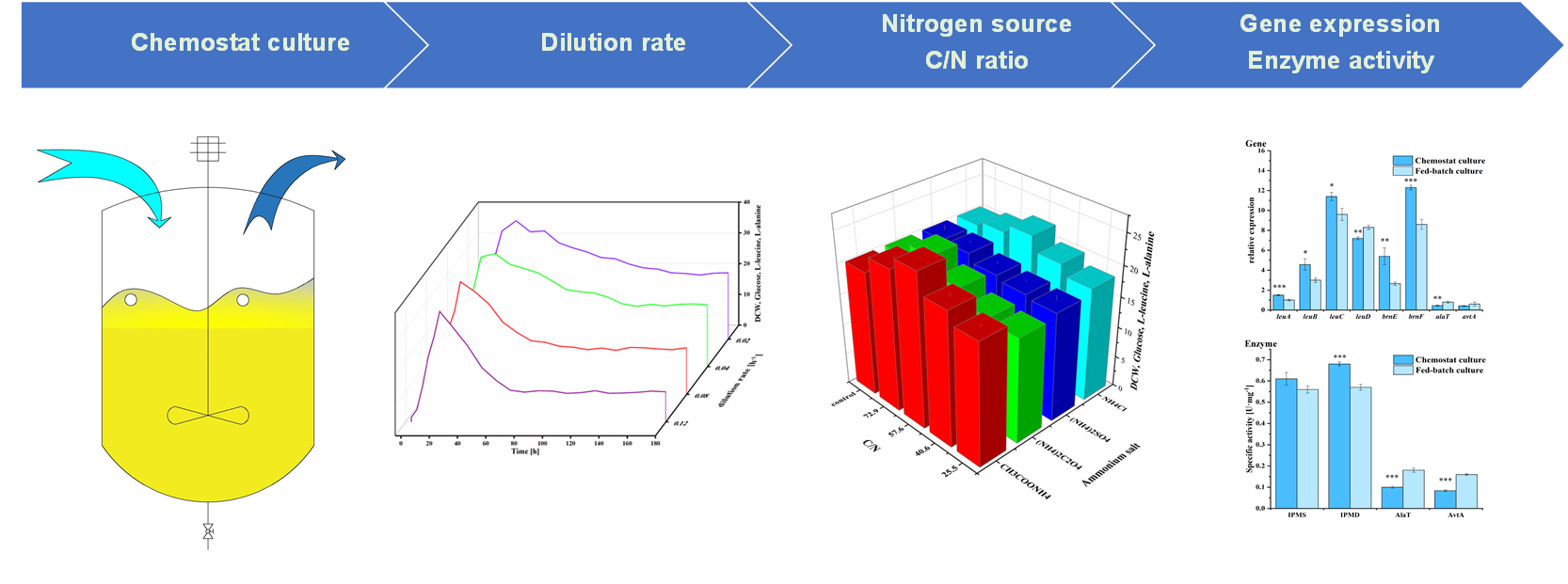

Supplement: Supplemental Material [file KBIE_A_1874693_SM1534.tif]
